# Loss of ING3 in the Prostate Leads to Activation of DNA Damage Repair Markers

**DOI:** 10.3390/cancers17061037

**Published:** 2025-03-20

**Authors:** Viktor Lang, Lisa Barones, ShiTing Misaki Hu, Fatemeh Hashemi, Karen Blote, Karl Riabowol, Dieter Fink

**Affiliations:** 1 Institute of Laboratory Animal Science, University of Veterinary Medicine Vienna, 1210 Vienna, Austria; 2 Departments of Biochemistry & Molecular Biology and Oncology, Cumming School of Medicine, University of Calgary, Calgary, AB T2N 4N1, Canada; 3 British Columbia Cancer Research Centre, Vancouver, BC V5Z 1L3, Canada

**Keywords:** *Ing3*, conditional knockout, prostate cancer, digital PCR

## Abstract

Background/Objectives: The inhibitor of growth family member 3 (ING3) acts as an epigenetic reader through physical interactions with histone-modifying enzymes and subsequent chromatin remodelling processes. It is involved in various cellular functions, such as cell cycle control, cell growth, and apoptosis. Although ING3 was assigned tumour suppressor candidate status in some types of cancers, including prostate cancer, some studies suggest it acts to promote growth. To address these contradictory reports regarding its role in the initiation and progression of prostate cancer, we specifically addressed the question of whether ablation of ING3 in the mouse prostate is sufficient to initiate malignant transformation of the prostate and support its (candidate) tumour suppressor status. Methods: To generate the prostate-specific *Ing3* knockout mouse, paternal inheritance of the PB-Cre4 transgene was used, while for the generation of a global knockout control, a female mouse harbouring the PB-Cre4 transgene was utilized. To determine the recombination efficiency of the Cre-LoxP system in the prostate at the *Ing3* locus, a duplex probe-based digital PCR assay capable of counting undisrupted *Ing3* copies was designed. The impact of DNA recombination on the protein level was investigated by immunohistochemical staining of prostate tissue samples. Results: In the prostate-specific knockout, digital PCR analysis revealed mosaic gene deletion. We found recombination efficiencies in the anterior, dorsolateral, and ventral prostate lobes ranging from approximately 15 to 30%. ING3 staining in the prostate was faint with no detectable differences in signal intensity between the knockout specimen and wild-type controls. This low ING3 expression in the prostate is consistent with observations of X-gal staining of an *Ing3*-LacZ reporter allele. Immunohistochemistry showed increased expression of DNA-damage-associated markers γH2AX and 53BP1. However, no gross anatomical abnormalities or prostate intraepithelial neoplasia (PIN) lesions in the prostate of tissue-specific knockout animals compared to wild-type controls were observed. Conclusions: Altogether, our data provide evidence that disruption of ING3 expression in prostate cells does not lead to malignant transformation and challenges the idea that ING3 acts primarily in a tumour-suppressive manner. Furthermore, this work supports the crucial role of ING3 in maintaining genomic stability, and we confirmed the embryonic lethal phenotype of homozygous *Ing3* null mice that is rescued by ectopic expression of ING3.

## Simple Summary

Prostate cancer is the most common cancer found in men, necessitating the study of genes implicated in the initiation and progression of this disease. The inhibitor of growth family member 3 (ING3) is an epigenetic regulator, whose role in prostate cancer is unknown. The aim of our study was to investigate the functional consequences of prostate-specific ablation of ING3 in mice. While we found normal prostate tissue histoarchitecture in the prostate-specific *Ing3* knockout mice, increased expression of DNA-damage-associated markers suggests a role for ING3 in maintaining genomic stability. Altogether, our data show that loss of *Ing3* does not lead to neoplastic transformation of the prostate.

## 1. Introduction

The inhibitor of growth 3 (ING3) belongs to a family of tumour suppressors encompassing five members (ING1–5), with the candidate tumour suppressor ING3 being the most evolutionarily distinctive [1]. The founding member, ING1, was discovered through differential screening of normal mammary epithelial cells and breast cancer cells [2]. Subsequently, ING2, 3, 4, and 5 were identified by applying bioinformatics approaches based upon sequence homology [3,4,5,6]. ING proteins share multiple highly conserved domains, such as a plant homeodomain (PHD), a lamin interaction domain, and a nuclear localization signal [1,7,8]. The PHD is an epigenetic reader module recognizing methylated lysine 4 residues of histone H3, marking transcriptional start sites [9,10].

As a stoichiometric component of the nucleosome acetyltransferase of histone H4 (NuA4)/TIP60 complex, ING3 is an evolutionarily conserved multi-subunit histone acetyltransferase (HAT), capable of adding acetyl groups to histone H4 and H2A N-terminal tails [11]. By recruiting the TIP60 complex to the chromatin, ING3 directs HAT activity to its specific substrates. Dynamic chromatin remodelling processes have major implications on gene-specific transcription processes. In general, acetylation of chromatin loosens the interaction between histones and DNA, thereby increasing chromatin accessibility and gene expression. Alterations in the maintenance and propagation of epigenetic modifications can give rise to the initiation and progression of various cancers [12]. Similarly to ING1, the candidate tumour suppressor ING3 can act as a negative growth regulator and enhance p53-dependent transcription [5]. For instance, ING3 enhances activity of the cyclin-dependent kinase inhibitor 1 (*CDKN1A*) promoter, providing a functional link to cell cycle regulation [13]. Differential expression of ING3 at the mRNA level in cell lines facing varying levels of reactive oxygen species may point towards a role in sensing harmful stimuli [14]. ING3 expression is also strongly enhanced after UV irradiation in a p53-independent manner, followed by activation of the Fas/caspase-8 pathway, thus linking genotoxic stress to apoptosis [15].

Considering the preceding factors, it is reasonable to predict an involvement of ING3 dysregulation in tumorigenesis and neoplastic growth. Downregulation of ING3 expression at the mRNA level has been linked to human head and neck cancers [16] and hepatocellular carcinoma [17], while reduced nuclear ING3 localization has been reported to be a key indicator of human cutaneous melanoma progression [18,19]. However, high ING3 expression levels have also been associated with a wide range of rapidly proliferating human tissues, including the small intestine, bone marrow, and epidermis [20]. In contrast to its putative tumour suppressor status, recent studies assign ING3 an oncogenic role in prostate cancer, based on a higher survival rate of human prostate cancer patients with lower ING3 levels [21]. This notion was supported by experimental data linking ING3 expression to induction of cellular proliferation in ex vivo benign prostate hyperplasia tissue [22]. Activation of the androgen receptor (AR) is an important mediator of prostate cancer pathogenesis. Upon binding androgenic hormones, the AR translocates into the nucleus and regulates the transcription of several target genes. The AR is a known substrate of TIP60 [23]. By physically interacting with both the AR and the TIP60 complex in the cytoplasm, ING3 directs HAT activity to the immediate vicinity of the AR, enhancing AR activation and nuclear translocation, resulting in the development and progression of prostate cancer [24].

Recently, we characterized an *Ing3* mutant mouse model in which a reporter cassette integrated into the *Ing3* locus, resulting in disruption and inactivation of the *Ing3* gene. Homozygous mutants were embryonically lethal, displaying growth retardation and impaired brain development, suggesting it has an important role in neuroectoderm development [25]. To investigate *Ing3* ablation in the mouse prostate, a conditional knockout mouse was generated. In brief, the knockout-first *Ing3*-tm1a (*Ing3*-LacZ) allele (Mouse Genome Informatics ID: 4432585) comprises an in-frame promoterless beta-galactosidase (LacZ)-tagged reporter cassette flanked by FLP recognition target (FRT) sites, and the critical exon 3 is flanked by LoxP sites. Successive exposure to FLP and Cre triggers the removal of the reporter cassette and the conversion from *Ing3*-LacZ to the tm1c allele and subsequently the excision of the critical exon 3 (conversion from tm1c to tm1d allele) [26,27]. Hereafter, *Ing3*^+^ represents the wild-type *Ing3* allele, *Ing3*^fl^ the *Ing3*-tm1c allele, and *Ing3*^Δ^ the Cre recombined *Ing3*-tm1d delta (knockout) allele.

In order to study the impact of *Ing3* inactivation in the prostate of adult mice, a prostate-specific *Ing3* knockout mouse model was generated. Additionally, a digital (d)PCR assay was established to confirm and assess recombination and *Ing3* deletion efficiency in the prostate. For calibration purposes and to confirm the embryonic lethal phenotype of ING3 null mice, a ubiquitous *Ing3* knockout line was generated. We also performed X-gal staining and immunohistochemistry on mouse prostate glands to assess wild-type ING3 expression levels and ING3 depletion in the knockout mice.

## 2. Materials and Methods

### 2.1. Animal Care and Husbandry

Mice were housed in a specific pathogen-free environment in compliance with FELASA recommendations [28] in individually ventilated cages (Eurostandard Type IIL; Tecniplast, Buguggiate, Italy) with adequate enrichment and nesting material. A regular mouse diet (ssniff Spezialdiäten, Soest, Germany) and water were provided ad libitum. The surrounding temperature and relative humidity were monitored and regulated (temperature: 22 °C ± 1; relative humidity: 50% ± 10). Procedures adhered to all federal, state, and local laws on animal experimentation (BMWFW-68.205/0049-WF/V/3b/2015 and BMWFW-68.205/0087-WF/V/3b/2015) and regulations issued by the Ethics and Animal Welfare Committee of the University of Veterinary Medicine Vienna, Vienna, Austria.

### 2.2. Generation of the Ing3-LacZ Reporter and Conditional Ing3 Knockout Animals

**Figure 1:**
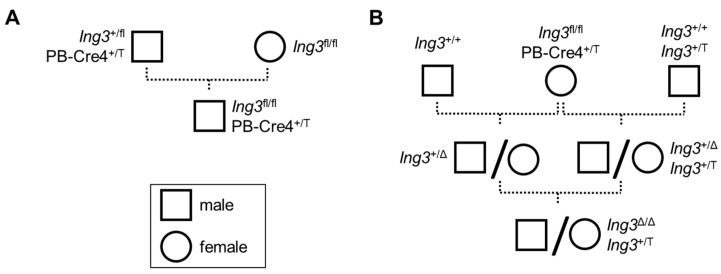
Mouse breeding scheme. (**A**) Generation of a prostate-specific conditional *Ing3* knockout mouse (*Ing3*^fl/fl^, PB-Cre4^+/T^). (**B**) Generation of a “ubiquitous *Ing3* knockout” mouse (*Ing3*^Δ/Δ^, *Ing3*^+/T^).

Germline-competent C57BL/6N embryonic stem cells harbouring the reporter-tagged *Ing3*^tm1a(EUCOMM)Wtsi^ knockout-first allele (*Ing3*-LacZ allele, EUCOMM clone EPD0028_2_H09) were injected into SWISS blastocysts. Chimaeras were bred to C57BL/6N animals for germline transmission to obtain the C57BL/6N-*Ing3*^tm1a(EUCOMM)Wtsi^/Biat knockout-first/*Ing3*-LacZ reporter animals. *Ing3*^fl/fl^ mice were obtained by FLP-mediated conversion of the *Ing3*-LacZ allele [27]. Prostate-specific *Ing3* knockout animals were obtained by mating *Ing3*^fl/fl^ mice with PB-Cre4 transgenic mice (Tg(Pbsn-cre)4Prb, JAX stock number 026662) through paternal transmission of the PB-Cre4 transgene (Figure 1A). For the generation of a ubiquitous *Ing3* knockout model, female PB-Cre4 transgenic mice were utilized as global Cre deleters. To produce viable “homozygous *Ing3* knockout” mice, ING3 expression was reconstituted by crossbreeding with the ING3 transgenic mouse line C57BL/6N-TgTn(sb-CAG-Ing3-P2A-eGFP)774.1Biat, hereafter referred to as the *Ing3*^T^ allele [25]. For the breeding scheme, see Figure 1B. Compound prostate-specific knockout animals harbouring one *Ing3*^fl^ and one mutated (disrupted) *Ing3*^m^ allele (B6(Cg)-Tyr^c−2J^ Tg(UBC-mCherry)1Phbs/J, JAX stock number 017614) described previously [25] were obtained by breeding male *Ing3*^fl/fl^, Pb-Cre^+/T^ mice with female *Ing3*^+/m^ mice. Table 1 summarizes the alleles of *Ing3* and transgenes used in this study.

### 2.3. PCR Genotyping

Ear notch biopsies and anterior (AP), dorsolateral (DLP), and ventral (VP) lobes of the prostate were collected and subjected to DNA extraction described previously [25]. The obtained DNA samples were processed according to the OneTaq Quick-Load protocol (New England Biolabs, Frankfurt, Germany; cat# M0486L) adjusted for a total reaction volume of 15 µL. Primers were designed using the free plasmid sequence editor tool ApE (version 2.0.50; https://jorgensen.biology.utah.edu/wayned/ape/ accessed on 30 August 2020) and synthesized as standard desalted DNA oligonucleotides (Sigma-Aldrich, Steinheim am Albuch, Germany).

PCR genotyping of the *Ing3*^T^ (CAG-Ing3-P2A-eGFP) allele and the *Ing3*^m^ (UbC-mCherry/*Ing3* mutant) allele was described previously [25]. Primers *Neo*-Forward (5′-CGCCTTCTATCGCCTTCTTG-3′) and *Ing3*-tm1a-Reverse (5′-GCTGGTTACGGCAATCAGAG 3′) amplify a 748 bp amplicon in mice carrying the *Ing3*-LacZ allele. PCR conditions were as follows: 95 °C for two minutes, 35 cycles of 95 °C for 30 s, 60 °C for 30 s, 68 °C for 60 s, and a final extension at 68 °C for two minutes. PB-Cre4 genotyping was performed with primers Cre-Forward (5′-CATTTCTGGGGATTGCTTATAACAC-3′) and Cre-Reverse (5′-TATTGAAACTCCAGCGCGGGCC-3′) that amplify a 434 bp amplicon. PCR amplification using the primers *Ing3*-Floxed-Forward (5′-TAGCAGCCATCCACTGAGG-3′) and *Ing3*-Floxed-Reverse (5′-GTTACGGCAATCAGAGCTGC-3′) yields a 250 bp band for the *Ing3*^+^ allele and a 413 bp band for the *Ing3*^fl^ allele. PCR conditions for Cre, *Ing3*^+^, and *Ing3*^fl^ genotyping were as follows: denaturation at 95 °C for two minutes, followed by 35 cycles of 95 °C for 30 s, 61 °C for 30 s, 68 °C for 30 s, and a final extension at 68 °C for two minutes. To detect the Cre-mediated conversion of the *Ing3*^fl^ allele into the *Ing3*^Δ^ allele (recombined knockout), the primers *Ing3*-Floxed-Forward and *Ing3*-Genomic-Reverse (5′-CAGGAAGCTCTAAACCAGTGC-3′) were used. The amplicon length for the *Ing3*^Δ^ allele is 293 bp, for the *Ing3*^fl^ allele 1108 bp, and for the wild-type allele 929 bp. PCR conditions were as follows: 95 °C for two minutes, 35 cycles of 95 °C for 30 s, 61 °C for 30 s, 68 °C for 60 s, and a final extension at 68 °C for two minutes.

### 2.4. Digital PCR

The DNA samples for dPCR calibration were obtained from ear biopsies from ubiquitous *Ing3* knockout animals (*Ing3*^Δ/Δ^, *Ing3*^+/T^), from heterozygous carriers (*Ing3*^+/Δ^), and homozygous floxed mice (*Ing3*^fl/fl^) and were harvested and processed as described above.

The primer pair and probe targeting the *Ing3*^+^, *Ing3*^fl^, and *Ing3*^m^ alleles were designed using Primer Express software (version 2.0; Applied Biosystems, Waltham, MA, USA). The melting temperatures and secondary structures of the primers were predicted with the software tool NetPrimer (http://www.premierbiosoft.com/netprimer/, accessed on 30 August 2020). The amplicon secondary structure folding was evaluated on the Mfold Web Server [30]. NCBI Primer-BLAST [31] was used to confirm the target specificity of the primers. Primers and the FAM-labelled ZEN/Iowa Black FQ double-quenched hydrolysis probe of the *Ing3* assay were synthesized at Integrated DNA Technologies (DNA Oligo and PrimeTime Eco qPCR Probe; Leuven, Belgium). *Ing3* was normalized to the single-copy nuclear gene beta-2-microglobulin (*B2m*). For copy number counting of *B2m*, a HydrolEasy probe was provided by PentaBase (Odense, Denmark). The probe was labelled with the reporter dye PentaYellow and the BHQ-1 quencher at its 5′ and 3′ ends, respectively, and contained five proprietary pentabases. Table 2 lists primer and probe sequences and corresponding amplicon lengths.

dPCR was performed on a QuantStudio 3D Digital PCR System (Applied Biosystems, Waltham, MA, USA). The concentration of template DNA was determined on an Eppendorf BioPhotometer (Eppendorf, Hamburg, Germany) equipped with an Implen LabelGuard Microliter Cell (Implen, Munich, Germany). A total of 80 ng of template DNA was applied per dPCR chip to fit into the narrow dynamic range of 200–2000 copies per microlitre of final reaction (QuantStudio 3D Digital PCR System User Guide; Applied Biosystems, Waltham, MA, USA). Reaction mixes were composed as outlined in Table 3 and loaded onto silicon chips (Applied Biosystems, Waltham, MA, USA; cat# A26316), encompassing 20,000 reaction wells, each with a capacity of 755 pl, using an automated chip loader (Applied Biosystems, Waltham, MA, USA; cat# 4482592). Chips were manually covered with immersion fluid and sealed with the provided lid according to the manufacturer’s instructions. Amplification was performed on a tilted flat block thermal cycler (GeneAmp PCR System 9700; Applied Biosystems, Waltham, MA, USA). The PCR cycling conditions included an initial incubation step at 96 °C for ten minutes, followed by 45 cycles of 98 °C for 30 s and 60 °C for two minutes and a final extension step at 60 °C for two minutes. End-point signals were read by the QuantStudio 3D Digital PCR Instrument (Applied Biosystems, Waltham, MA, USA; cat# 4481097) and curated on the QuantStudio 3D AnalysisSuite Cloud Software (version 3.1.6-PRC-build2; Applied Biosystems, Waltham, MA, USA).

Instrument readouts including Poisson distribution parameters and quality assessment metrics are provided in the supplementary data (Tables S7 and S8) in compliance with the “Minimum Information for Publication of Quantitative Digital PCR Experiments” guidelines [32]. Samples that did not fit into the dynamic range of 200–2000 copies per microlitre were omitted from statistical analysis. Statistical analyses and plotting of the results were performed using Prism (version 5.00; GraphPad Software, San Diego, CA, USA).

### 2.5. SDS-PAGE and Western Blotting

Whole-cell lysates were prepared for SDS-PAGE and immunoblotting (20 µg protein loaded per lane) as previously described [25] using primary anit-ING3 antibody (1:200; Merck, Darmstadt, Germany; cat# MABC1185; this is the commercially available anti-ING3 monoclonal antibody clone 2A2 (Riabowol Laboratory, University of Calgary, Calgary, AB, Canada)) and anti-vinculin antibody (1:200; Santa Cruz Biotechnology, Dallas, TX, USA; cat# sc-25336).

### 2.6. Immunohistochemical Staining and Immunofluorescence

The prostates were harvested and placed in 10% formalin (VWR International, Radnor, PA, USA; cat# 9713.1000) overnight, stored in 70% ethanol, and embedded in paraffin blocks. Kidney, liver, brain, and periprostatic soft tissue samples of a male wild-type mouse were included as positive controls (Figure S1) for ING3 expression.

Paraffin blocks were cut into 5 µm sections (HM 325 Rotary Microtome; Thermo Scientific, Waltham, MA, USA), and the paraffin ribbons were placed in a water bath at 37 °C and mounted onto adhesive-coated slides (VWR International, Radnor, PA, USA; cat# 48311-703). After air-drying overnight at room temperature, tissues were deparaffinized by three rounds of incubation in xylene for 15 min. Rehydration was performed in consecutive baths of 100% ethanol, 100% ethanol in a different vessel, 95% ethanol, 70% ethanol, and Milli-Q water (Merck, Darmstadt, Germany) for five minutes each. The slides were fully submerged in antigen retrieval buffer (10 mM Tris-base, 1 mM EDTA, 0.05% Tween 20, pH adjusted to 9.0), and the buffer was brought to a boil in a microwave and kept at 95 °C for another 20 min. After cooling, the samples were washed with Tris-buffered saline with Tween 20 (TBST) washing buffer (150 mM NaCl, 20 mM Tris-base, pH 7.6, supplemented with 0.05% Tween 20) four times for four minutes and then incubated with Dako REAL™ Peroxidase-Blocking Solution (Dako, Agilent Technologies, Santa Clara, CA, USA; cat# S202386-2) for 15 min. For immunofluorescence, the peroxidase-blocking step was omitted. After washing four times for four minutes with TBST, the sections were treated with blocking solution (TBST supplemented with 2% BSA) for 30 min at room temperature. The samples were washed once for two minutes with TBST and then incubated with primary anti-53BP1 (1:2000; Abcam, Cambridge, UK; cat# ab21083), anti-cleaved caspase-3 (1:400; Cell Signaling Technology, Danvers, MA, USA; cat# 9661), anti-Ki67 (1:500; Abcam, Cambridge, UK; cat# ab15580), anti-γH2AX (1:100; Abcam, Cambridge, UK; cat# ab26350), or anti-ING3 monoclonal antibody clone 2A2 (1:100; in-house, Riabowol Laboratory, University of Calgary, Calgary, AB, Canada) diluted in staining buffer (150 mM NaCl, 20 mM Tris-base, pH 7.6, supplemented with 1% BSA) overnight at 4 °C. Negative controls were treated with staining buffer instead of the primary antibody. The primary antibody was removed by four consecutive washes with TBST for four minutes each. For immunohistochemistry, the sections were incubated with horseradish peroxidase (HRP)-labelled polymer anti-mouse reagent (Dako, Santa Clara, CA, USA; cat# K4000) or anti-rabbit (1:1000; Merck, Darmstadt, Germany; cat# 12-348) for one hour at room temperature. After washing four times in TBST for four minutes each, the sections were treated with 3,3′-Diaminobenzidine (DAB) reagent (1 mL DAB substrate supplemented with one drop of DAB chromogen (Dako, Santa Clara, CA, USA; cat# K3468)) and incubated for approximately two minutes until a visible change in colour occurred. The samples were washed with Milli-Q water and then counterstained with hematoxylin (Sigma-Aldrich, Saint Louis, MO, USA; cat# GHS232) for five minutes. The slides were rinsed with warm tap water (approximately 37 °C) for three minutes, submerged in TBST for one minute, and washed with warm Milli-Q water for one minute. Dehydration was performed by dipping the slides 15 times per vessel in a graded series of alcohol (70%, 95%, and two different vessels containing 100% ethanol) and finally two different vessels filled with xylene. Mounting solution (Biocare Medical, Concord, CA, USA; cat# EM897L) was applied, and coverslips were placed on the specimens.

For immunofluorescence, the sections were incubated with AF488-conjugated secondary antibody, specifically Goat Anti-Mouse IgG H&L (Abcam, Cambridge, UK; cat# ab150113) or Goat Anti-Rabbit IgG H&L (Abcam, Cambridge, UK; cat# ab150077), at a 1:1000 dilution for one hour. Following incubation, the sections were washed three times, five minutes each, with TBST. Nuclear staining was performed using SlowFade Diamond Antifade Mountant with DAPI (Thermo Scientific, Waltham, MA, USA; cat# S36973) as the mounting solution.

Staining was confirmed through brightfield microscopy (CX41 Upright Microscope; Olympus, Tokyo, Japan) or with a fluorescence microscope (AxioObserver; Zeiss, Oberkochen, Germany). Images were acquired with QCapture (software version 18.3; Teledyne QImaging, Surrey, BC, Canada) in combination with a digital camera (Q-Color5 Digital Imaging System; Olympus, Tokyo, Japan) and edited using the open-source image processing software GIMP (version 2.10.18).

### 2.7. X-Gal Staining of Cryosections

For X-gal staining, harvested prostate tissues of *Ing3*-LacZ heterozygous mice and wild-type control mice were placed into cryomolds (Tissue Tek Cryomolds Standard 25 mm × 20 mm × 5 mm, and Tissue Tek Cryomolds Intermediate 15 mm × 15 mm × 5 mm; Sakura, Torrance, CA, USA; cat# 25608-916 and 25608-924), covered with optimal cutting temperature compound (Tissue Tek O.C.T. compound; Sakura, Torrance, CA, USA; cat# 4583), and immediately submerged and frozen in hexane (Sigma-Aldrich, Saint Louis, MO, USA; cat# 296090-1L) placed on dry ice. The frozen samples were wrapped in aluminium foil and stored at −80 °C.

For sectioning, the samples were placed in the Cryostat (CryoStar NX70; Thermo Scientific, Waltham, MA, USA) at −20 °C for one to two hours. Then, 5 µm sections were cut and placed on slides (two per slide; Menzel Gläser Superfrost Ultra Plus; Thermo Scientific, Waltham, MA, USA; cat# J4800AMNZ) and air-dried for 30 min. The slides were stored at −20 °C for two to three hours prior to X-gal staining. For X-gal staining, the slides were removed from the −20 °C freezer and air-dried for 20 min. Each sample was circled with Pap-Pen liquid (Super PAP Pen Liquid Blocker Mini; Science Services, Munich, Germany; cat# N71312) and dried for 15–20 s. The slides were placed in 0.2% (*w*/*v*) glutaraldehyde (Glutaraldehyde 25%; Sigma-Aldrich, Saint Louis, MO, USA; cat# G5882-50ML) in PBS (8.00 g of NaCl, 0.20 g of KCl, 1.78 g of Na_2_HPO_4_·2H_2_O (Carl Roth, Karlsruhe, Germany; cat# 4984.2), and 0.24 g of KH_2_PO_4_ (Sigma-Aldrich, Saint Louis, MO, USA; cat# P5655-100G) dissolved in 1 L of Milli-Q water, pH 7.4) for ten minutes at 4 °C and washed three times in PBS at room temperature for five minutes each. Each slide was tapped on a paper towel to remove the remaining PBS and placed into a 37 °C incubator. Next, 300 µL of X-gal staining solution (80 µL of stock solution (25 mg/mL of X-gal (Sigma-Aldrich, Saint Louis, MO, USA; cat# B4252-50MG) in dimethyl sulfoxide (DMSO; Sigma-Aldrich, Saint Louis, MO, USA; cat# D2650)) diluted in 2 mL of PBS containing 5 mM ferricyanide (Sigma-Aldrich, Saint Louis, MO, USA; cat# 244023-100G), 5 mM ferrocyanide (Sigma-Aldrich, Saint Louis, MO, USA; cat# P3289-100G), and 2 mM MgCl_2_ 6H_2_O (Mallinckrodt Baker, Blanchardstown, Dublin, Ireland; cat# 2444-01)) was dripped on each sample. The slides remained in the incubator overnight (approximately 19 h).

The stained samples were briefly rinsed once in PBS, then washed in PBS for ten minutes and washed two times in Milli-Q water for five minutes each. After counterstaining with nuclear fast red solution (Sigma-Aldrich, Saint Louis, MO, USA; cat# N3020) for five minutes, the slides were briefly rinsed and washed in Milli-Q water for two minutes. The slides were covered with cover slips (Cover glasses; VWR, Radnor, PA, USA; cat# 631-0127) and Fluoromount G (Sigma-Aldrich, Saint Louis, MO, USA; cat# F4680-25ML) and air-dried for at least 30 min. Images at magnifications of 100×, 200×, and 400× were taken and evaluated with the Olympus BX41 microscope equipped with a Leica DFC420 C camera and Leica LAS software (version 3.1; Leica Microsystems, Wetzlar, Germany).

### 2.8. Whole-Mount X-Gal Staining

Prostate tissues were harvested from *Ing3*-LacZ heterozygous and wild-type control male mice, placed in PBS, and immediately proceeded with the staining procedure.

Tissues were transferred to 0.2% (*w*/*v*) glutaraldehyde-fixative (0.2% glutaraldehyde, 2 mM MgCl_2_·6H_2_O, 5 mM EGTA (Sigma-Aldrich, Saint Louis, MO, USA; cat# E4378-10G) in sodium phosphate buffer (SPB; 5.3 mL of solution A (200 mM NaH_2_PO_4_·2H_2_O (Sigma-Aldrich, Saint Louis, MO, USA; cat# 71500-250G)) and 94.7 mL of solution B (200 mM Na_2_HPO_4_·2H_2_O diluted with Milli-Q water to 200 mL, pH 8.0))) for 90 min at 4 °C. Afterwards, the samples were washed three times in basis staining buffer (BSB; 2 mM MgCl_2_·6H_2_O, 0.01% (*w*/*v*) Na-deoxycholate (Sigma-Aldrich, Saint Louis, MO, USA; cat# D6750-10G), 0.02% (*v*/*v*) Nonidet P-40 (Sigma-Aldrich, Saint Louis, MO, USA; cat# I3021), 5 mM EGTA in SPB, pH 8.0) for 30 min and submersed in X-gal staining solution (80 µL of X-gal stock solution in 2 mL of BSB containing 10 mM ferricyanide and 10 mM ferrocyanide) for five to eight hours (depending on the staining solution, which was re-used up to four times with a clear reduction in staining intensity). The samples were covered with aluminium foil and placed in a 37 °C shaking incubator. Stained samples were washed two times in basis staining buffer for 30 min and submerged into 4% formaldehyde (buffered aqueous solution, 0.5–1.5% methanol; VWR, Radnor, PA, USA; cat# 9713.1000) for nine to ten hours at 4 °C. Fixed samples were washed briefly in 70% ethanol and placed into fresh 70% ethanol for storage at 4 °C. Images were taken and evaluated with the Leica MZ 16 FA microscope (Leica Microsystems, Wetzlar, Germany) and a Leica DFC310 FX camera utilizing the Leica LAS AF software (version 3.3; Leica Microsystems, Wetzlar, Germany).

## 3. Results

### 3.1. Animal Breeding

The breeding strategy to obtain prostate-specific *Ing3* knockout animals (paternal inheritance of PB-Cre4 allele) is shown in Figure 1A. To confirm recombination and quantify recombination efficiency at the *Ing3* locus in the prostatic lobes, we established a dPCR calibration method that allows for absolute measurement of recombination fraction in the tissues. To obtain the fully recombined *Ing3* knockout at the endogenous locus needed for dPCR calibration, systemic Cre recombination was achieved by maternal inheritance of PB-Cre4 and ectopic ING3 expression as shown in Figure 1B. A comprehensive list of all animals included in the experiments is provided in the supplementary data (Tables S1–S6).

**Figure 2:**
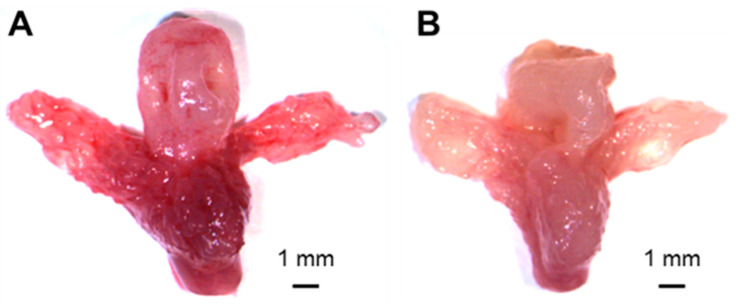
Representative image of prostate gross anatomy in age-matched mice. (**A**) Prostate-specific *Ing3* knockout (*Ing3*^fl/fl^, PB-Cre4^+/T^ (*n* = 22, 12–87-week range)) and (**B**) wild-type control (*Ing3*^fl/fl^, PB-Cre4^+/+^ (*n* = 17, 12–74-week range)). Scale bars are shown at the right bottom of each image.

In the prostate-specific *Ing3* knockout animals, no signs of abnormalities with regard to prostate size and appearance or neoplastic transformation were detected upon gross anatomic inspection of 22 individual animals ranging in age from 12 to 87 weeks compared to wild-type controls (Figure 2).

### 3.2. Immunohistochemistry of Prostate Tissues

**Figure 3:**
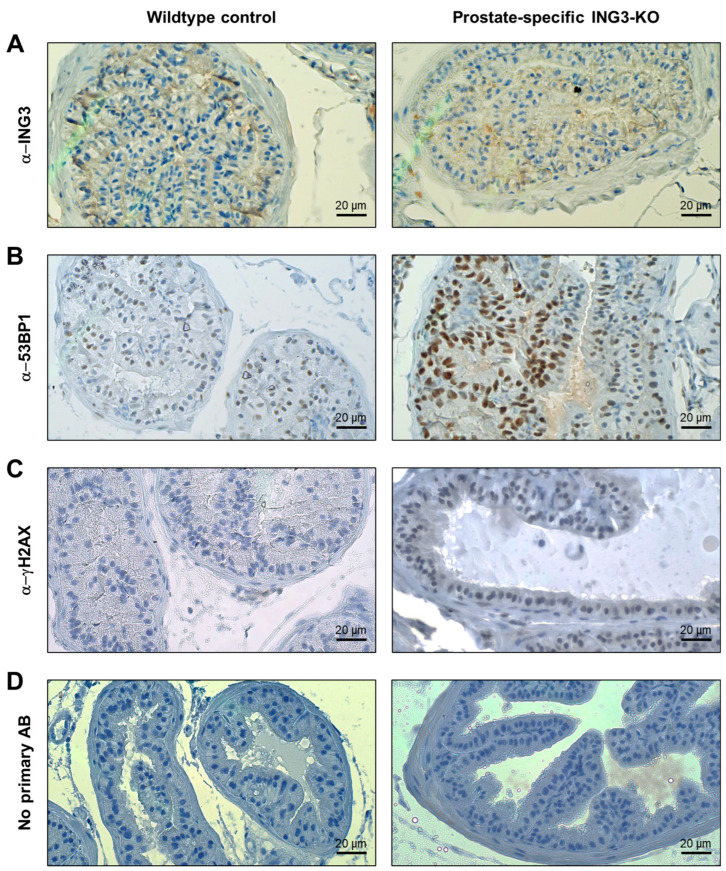
Representative images of immunohistochemical staining of prostatic lobes of age-matched prostate-specific *Ing3* knockouts and wild-type controls. Staining is shown for (**A**) ING3, (**B**) 53BP1 (**C**) γH2AX, and (**D**) negative control with omitted primary antibody. Left-side panel: *Ing3*^fl/fl^, PB-Cre4^+/+^ (*n* = 3); right-side panel: *Ing3*^fl/fl^; PB-Cre4^+/T^ (*n* = 3). Magnification: 400×. Scale bars are shown at the bottom right of each image.

For ING3 expression assessment in the prostate-specific *Ing3* knockout model, the dorsolateral prostatic lobe of an *Ing3*^fl/fl^, PB-Cre4^+/T^ mouse was compared with an *Ing3*^fl/fl^, PB-Cre4^+/+^ wild-type control mouse (Figure 3A). The overall staining intensity was faint, and thus it was not feasible to draw qualitative or quantitative conclusions on the recombination success based on IHC staining. In sections where the primary antibody was omitted, no ING3 staining signal was detected (Figure 3D). These results are consistent with staining by IHC in the human prostate, where weak staining was seen previously in a small subset (~9%) of cells [20]. Of note, ING3 could not be detected in whole-cell lysates from wild-type murine prostates by western blot analysis (Figure S2). γH2AX was slightly increased, while nuclear 53BP1 was strongly increased in the prostates of *Ing3*^fl/fl^, PB-Cre4^+/T^ mice compared to wild-type controls (Figure 3B,C and Figure S3), whereas no discernible differences were observed in cleaved caspase-3 and Ki67 staining (Figure S4). In addition, prostate intraepithelial neoplasia (PIN) was not detected in prostate-specific *Ing3* knockout mice.

### 3.3. X-Gal Staining of Ing3-LacZ Reporter Mice

**Figure 4:**
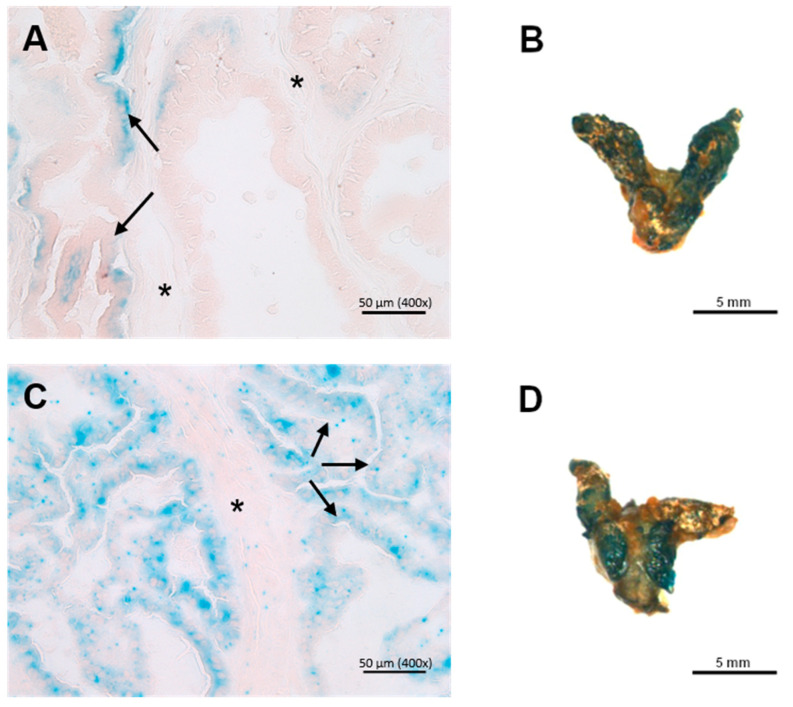
Representative X-gal staining of (**left**) cryosections of the dorsal prostate and (**right**) whole-mount prostates of age-matched animals. (**A**,**B**) Wild-type (*n* = 3) and (**C**,**D**) *Ing3*-LacZ transgenic mice (*n* = 3). Arrows indicate adenocytes of dorsal prostate; asterisks indicate connective tissue. Scale bars and/or magnifications are shown at the bottom right of each image.

ING3 expression evaluation by X-gal staining of the prostate in cryosections showed faint staining in wild-type controls with positive basal parts of the epithelium (adenocytes) and very low-grade staining intensity of the connective tissue compared with the *Ing3*-LacZ reporter animals, where the staining was homogenous throughout the epithelial lining (Figure 4A,C). Evaluation of whole-mount staining of the prostate was inconclusive due to the similar macroscopic staining intensity of the wild-type controls (Figure 4B,D).

### 3.4. Digital PCR Calibration and Recombination Efficiency Assessment in the Prostate

**Figure 5:**
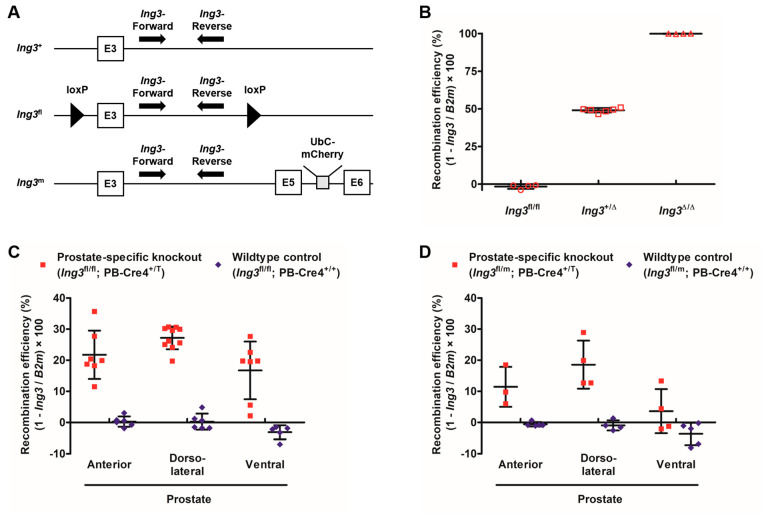
Recombination efficiency assessment via dPCR. (**A**) Schematic view of *Ing3* alleles targeted by dPCR primers. Primer annealing sites are depicted by arrows. (**B**) Calibration of the dPCR assay using *Ing3*^fl/fl^ (*n* = 4); *Ing3*^+/Δ^ (*n* = 6); and *Ing3*^Δ/Δ^, Ing3^+/T^ (*n* = 4) mice. (**C**) Recombination efficiency in the prostate-specific *Ing3* knockout (*Ing3*^fl/fl^, PB-Cre4^+/T^: AP (*n* = 7), DLP (*n* = 10), and VP (*n* = 7); *Ing3*^fl/fl^, PB-Cre4^+/+^: AP (*n* = 6), DLP (*n* = 6), and VP (*n* = 5)). (**D**) Recombination efficiency in the prostate of compound heterozygotes (*Ing3*^fl/m^, PB-Cre4^+/T^: AP (*n* = 3), DLP (*n* = 4), and VP (*n* = 4); *Ing3*^fl/m^, PB-Cre4^+/+^: AP (*n* = 5), DLP (*n* = 4), and VP (*n* = 5)). Each symbol represents one animal. Standard deviation is indicated by error bars.

The *Ing3*^fl^, *Ing3*^+^, and *Ing3*^m^ alleles are shown in Figure 5A. The outcome of the three-point calibration is shown in Figure 5B. The recombination values for the three distinct genotypes *Ing3*^fl/fl^ (−2% ± 1); *Ing3*^+/Δ^ (49% ± 1); and *Ing3*^Δ/Δ^, *Ing3*^+/T^ (100% ± 0) were as expected (0%, 50%, and 100%, respectively). Values below 0% are method-inherent fluctuations of measurements.

To evaluate Cre-mediated recombination in the prostate-specific knockout compared to age-matched wild-type animals, DNA samples from anterior (AP), dorsolateral (DLP), and ventral prostatic lobes (VP) were analysed. The Cre-mediated recombination efficiencies of the distinct prostate lobes of *Ing3*^fl/fl^, PbCre4^+/T^ mice were AP 22% ± 8, DLP 27% ± 4, and VP 17% ± 9 compared to *Ing3*^fl/fl^, PbCre4^+/+^ mice with AP 0% ± 2, DLP 0% ± 3, and VP −3% ± 2, as shown in Figure 5C. To assure full *Ing3* deletion in a single prostate cell, Cre recombination was also analysed in double mutants carrying one *Ing3*^fl^ and one *Ing3*^m^ allele. Recombination efficiencies in *Ing3*^fl/m^, PbCre4^+/T^ mice were AP 11% ± 6, DLP 19% ± 8, and VP 4% ± 7 compared to *Ing3*^fl/m^, PbCre4^+/+^ mice with AP −1% ± 1, DLP −1% ± 2, and VP −4% ± 4, as shown in Figure 5D.

## 4. Discussion

No differences in the anatomy of the prostate between prostate-specific knockout and wild-type animals support that loss of ING3 does not lead to neoplastic changes and has no influence on the growth of the prostate. Due to the poor signal-to-noise ratio in the IHC in the wild-type prostate, we were not able to detect ING3 ablation in the prostate-specific knockout. This is also consistent with background LacZ staining in the wild-type control showing no difference to the *Ing3*-LacZ reporter line in whole-mount prostate staining and western blot analysis of wild-type prostate. High ING3 expression was detected in the kidney, moderate expression in peripheral neurons in the prostate, and weak diffuse expression in the liver. This is consistent with previous reports on low ING3 expression in the prostate [20,33]. Nevertheless, the digital PCR showed that full recombination occurred in 20–30% of the cells of the prostate. To exclude the minimal possibility that only one floxed allele of a cell is recombined by Cre recombinase and one allele is left intact, we intercrossed the conditional *Ing3*^fl/fl^ with the *Ing3* mutant (*Ing3*^+/m^) [25] to provide one conditional floxed and one full mutated allele (*Ing3*^fl/m^). The recombination efficiency is approximately twice as high in *Ing3*^fl/fl^, PB-Cre4^+/T^ compared to *Ing3*^fl/m^, PB-Cre4^+/T^ mice, as expected (Figure 5C,D).

It was reported that the Cre-activity varies between the anterior, dorsolateral, and ventral prostate lobes when the PB-Cre4 strain was crossed to a *lacZ* reporter strain [29], as we have also demonstrated in this work. Also, Cre recombination in general is dependent on the chromosomal locus, distance and flanking sequences of the LoxP sites, and Cre expression levels within individual cells [34]. We have noticed an overall slight increase in recombination efficiency over time (a maximum of approximately 35% in the anterior prostate in 36-week-old mice), as shown in Figure S5. Nevertheless, this is far from full recombination that was suggested by LacZ staining of 32-week-old mice when characterizing the PB-Cre4 transgenic mouse line [35], supporting the influence of the locus and/or the detection method. Therefore, the dPCR as demonstrated within this work is the method of choice for evaluation of absolute recombination efficiency in Cre recombined conditional knockout alleles when no phenotype was observed and the IHC is not applicable.

Upon gross anatomical inspection of 22 prostate-specific *Ing3* knockouts aged 12–87 weeks, there was no evidence of prostate tumorigenesis or neoplastic transformation (Figure 2), also supported by no changes in Ki67 proliferation stainings (Figure S4). In contrast, deletion of the classical gatekeeper tumour suppressor gene phosphatase and tensin homologue (*Pten*), which dephosphorylates phosphatidylinositol-(3,4,5)-trisphosphate and acts as a negative regulator of the protein kinase B signalling pathway, leads to invasive adenocarcinoma with a reported cumulative incidence of 100% within 29 weeks of age [36]. Moreover, mosaic deletion of E-cadherin (*Cdh1*) causes PIN lesion formation, demonstrating that genetic aberrations in a fraction of prostate cells are sufficient to induce oncogenic transformation [37]. The same tissue-specific Cre deleter mouse line was used in this study. Based on demonstrated partial recombination, we conclude that in prostate tissue, *Ing3* is not a gatekeeper tumour suppressor comparable to haploinsufficient *Pten*.

Although most studies report the assumption that *Ing3* acts as a negative regulator of cell growth and proliferation, hence the name inhibitor of growth 3, overexpression of ING3 was recently associated with enhanced cellular proliferation in ex vivo prostate tissue cultures [22]. This is consistent with our previous finding that loss of ING3 expression results in growth restrictions during embryonic development [25]. Recently, a novel splice variant lacking the PHD domain was identified in human prostate cancer samples. Ectopic overexpression of ING3Δex11 (loss of PHD) in LNCaP-derived 3D spheroids led to morphological changes associated with tumour invasiveness and increased expression of epithelial mesenchymal transition (EMT) markers/transcription factors, suggesting an oncogenic role. Mechanistically, wild-type ING3 could exert tumour-suppressive functions in the nucleus via chromatin-dependent induction of EMT-suppressive genes. Subsequent loss of the chromatin-interacting PHD domain could release the brake on EMT-promoting factors [38]. In human MCF7 mammary carcinoma cells, loss of H3K4me3 binding through PHD-mutated ING3 leads to decreased apoptosis upon DNA damage, supporting wild-type ING3 tumour-suppressive function [39]. Furthermore, wild-type ING3 was shown to be essential for ATM activation that subsequently mediates phosphorylation of NBS1, which is necessary for recruitment of the major mediators of the DNA damage response [40]. We observed increased staining intensity of 53BP1 and γH2AX upon prostate-specific disruption of wild-type *Ing3*, suggesting a response to DNA damage, thus positioning wild-type ING3 as a caretaker in maintaining genomic integrity. This is consistent with the observation that ING3 suppresses the activation of endogenous retroviruses to maintain genomic stability [41]. Considering these data, overexpression of wild-type *Ing3* compared to the PHD negative variant in a double knockout of *Pten* and *p53* [42,43] should resolve the hypothesis of the oncogenic role of *Ing3*. Furthermore, speculating that a triple knockout of *Pten*, *p53*, and *Ing3* results in increased aggressiveness of the tumour metastasis will support the hypothesis of the tumour suppressor function of *Ing3* needing a second or even third hit. Overall, loss of ING3 does not lead to neoplastic transformation but rather to activation of DNA damage response genes, as demonstrated in this work.

## 5. Conclusions

ING3 is still referred to as a candidate tumour suppressor based on initial studies performed in cancer cell lines and immunohistochemistry in normal and cancer tissues. Recently, it was attributed oncogenic behavior in prostate cancer in several studies, and therefore, its role has not yet been clarified. Within this study, we have generated a prostate-specific *Ing3* knockout mouse and demonstrated by dPCR the disruption of the *Ing3* locus. Furthermore, activation of DNA damage repair markers also supports the loss of ING3 expression. We have not noticed any gross anatomical changes of the prostate or PIN lesion formation and concluded that ING3 is not a tumour suppressor comparable to PTEN that clearly results in visible malignant neoplastic transformation. Further, ING3 knockout and gain-of-function studies in mice in combination with loss of PTEN and a third hit will clarify its tumour suppressor and/or oncogenic function.

## Figures and Tables

**Table 1:** Mouse alleles of *Ing3* and transgenes used in this study.

Allele Designation	Description	Reference
*Ing3* ^+^	Wild-type allele	NCBI Gene: 71777
*Ing3*-LacZ	Targeted knockout-first; reporter-tagged insertion with conditional potential (*Ing3*^tm1a(EUCOMM)Wtsi^/Biat)	MGI: 4432585 This work
*Ing3* ^fl^	Derived from *Ing3*-LacZ through FLP-mediated recombination; conditional allele (*Ing3*-tm1c)	This work
*Ing3* ^Δ^	Derived from *Ing3*^fl^ through Cre-mediated recombination; null allele (*Ing3*-tm1d)	This work
*Ing3* ^m^	Insertional mutant; disruption of *Ing3* expression by mCherry reporter cassette (Tg(UBC-mCherry)1Phbs)	MGI: 5296812 [25]
*Ing3* ^T^	Transgenic; ectopic overexpression of *Ing3* (TgTn(sb-CAG-Ing3-P2A-eGFP)774.1Biat)	[25]
PB-Cre4	Transgenic; Cre expression in prostate epithelial cells (Tg(Pbsn-cre)4Prb)	MGI: 2385927 [29]

**Table 2:** dPCR primer and probe sequences.

Target Gene	NCBI Gene ID	Primer/Probe Designation	Primer Sequence (5′-3′)	Amplicon Length (bp)
*Ing3*	71777	*Ing3*-Forward	CATTGGGACCCTCTAGGAGAGAT	82
		*Ing3*-Reverse	GCCCCCAAGTCCCTCATAA	
		*Ing3*-Probe	/56-FAM/TTACGTAGA/ZEN/TACCT GGATATGGAGTGAGGGCA/3IABkFQ/	
*B2m*	12010	*B2m*-Forward	CTCAGAAACCCCTCAAATTCAAGTA	96
		*B2m*-Reverse	GGCGGGTGGAACTGTGTTAC	
		*B2m*-Probe	/PentaYellow/CTCACGCCACCCAC CGGAGAATG/BHQ-1/	

**Table 3:** dPCR reaction mix.

Component	Volume Per Reaction (µL)	Stock Concentration	Final Concentration
QuantStudio 3D Digital PCR Master Mix v2	9.00	2 ×	1 ×
*Ing3*-Forward	0.36	10 µM	0.2 µM
*Ing3*-Reverse	0.36	10 µM	0.2 µM
*Ing3*-Probe	0.36	10 µM	0.2 µM
*B2m*-Forward	0.36	10 µM	0.2 µM
*B2m*-Reverse	0.36	10 µM	0.2 µM
*B2m*-Probe	0.36	10 µM	0.2 µM
H_2_O	1.84	-	-
Template DNA	5.00	-	-
Total volume	18.00		

## Data Availability

Data are contained within the article or supplementary material.

## Supplementary Materials

The following supporting information can be downloaded at: https://www.mdpi.com/article/10.3390/cancers17061037/s1. Figure S1: Representative images of ING3 staining in murine tissues. Figure S2: Western blot analysis of ING3 in wild-type mouse tissues. Figure S3: Representative immunofluorescence images of the prostate. Figure S4: Representative images of cleaved caspase 3 and Ki67 staining of prostatic lobes of age-matched prostate-specific *Ing3* knockouts and wild-type controls. Figure S5: Recombination efficiency in the prostate lobes in prostate-specific knockout and wild-type *Ing3* mice as a function of age. Table S1: Animals used for dPCR calibration. Table S2: Animals used for gross anatomic inspection and prostate-specific recombination efficiency assessment by dPCR. Table S3: Animals used for gross anatomic inspection, immunohistochemistry, and immunofluorescence. Table S4: Animals used for gross anatomic inspection only. Table S5: Animals used for X-gal staining of cryosections. Table S6: Animals used for X-gal staining of whole-mount tissues. Table S7: Instrument readout for dPCR calibration. Table S8: Instrument readout for dPCR recombination experiment.

## Abbreviations

The following abbreviations are used in this manuscript:

AP	Anterior prostate
AR	Androgen receptor
ATM	Ataxia-telangiectasia mutated
*B2m*	Beta-2-microglobulin
BSA	Bovine serum albumin
BSB	Basis staining buffer
*Cdh1*	E-cadherin
*CDKN1a*	Cyclin-dependent kinase inhibitor 1A
Cre	Cyclization recombinase
DAB	3,3′-Diaminobenzidine
DAPI	4′,6-diamidino-2-phenylindole
DLP	Dorsolateral prostate
DMSO	Dimethyl sulfoxide
dPCR	Digital polymerase chain reaction
EDTA	Ethylenediaminetetraacetic acid
EGTA	Ethylene glycol-bis(2-aminoethylether)-N,N,N′,N′-tetraacetic acid
EMT	Epithelial mesenchymal transition
FLP	Flippase
FRT	Flippase recognition target
HAT	Histone acetyltransferase
HRP	Horseradish peroxidase
IHC	Immunohistochemistry
ING3	Inhibitor of growth 3
*lacZ*	β-galactosidase gene
LoxP	Locus of X-over P1
mRNA	Messenger RNA
NBS1	Nibrin
NuA4	Nucleosome acetyltransferase of histone H4
PB	Probasin
PBS	Phosphate-buffered saline
PCR	Polymerase chain reaction
PHD PIN	Plant homeodomain Prostatic intraepithelial neoplasia
*Pten*	Phosphatase and tensin homologue
SPB	Sodium phosphate buffer
TBST	Tris-buffered saline with Tween 20
VP	Ventral prostate
X-gal	5-Bromo-4-chloro-3-indolyl β-D-galactopyranoside
