# Supplementary material for: Loss of ING3 in the Prostate Leads to Activation of DNA Damage Repair Markers
Source: Cancers (Basel). 2025 Mar 20;17(6):1037. doi: 10.3390/cancers17061037 (PMC11940784; doi:10.3390/cancers17061037)
Supplement: Supplementary file 1 [file cancers-17-01037-s001.zip › cancers-3473949-supplementary.pdf]

Supplementary material

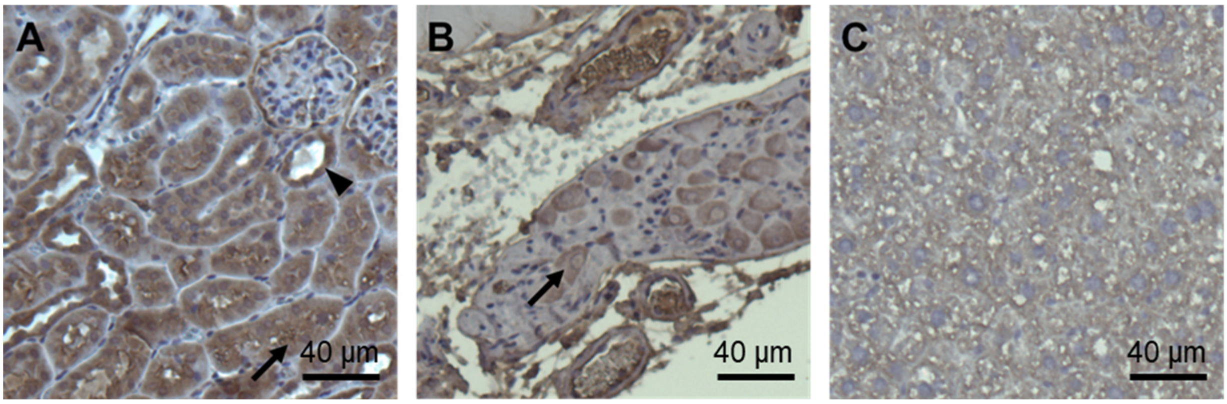

Figure S1. Representative images of ING3 staining in murine tissues. Strong staining of the (**A**) kidney proximal (arrow) and distal (arrowhead) tubules of the renal cortex. Moderate staining intensity in (**B**) neuron cell body (arrow) of a ganglion in periprostatic soft tissue and weak diffuse staining in (**C**) liver sections. Kidney, prostate tissue, and liver were extracted from a male wild type mouse. Magnification 200×. Scale bars are shown at the right bottom of each image.

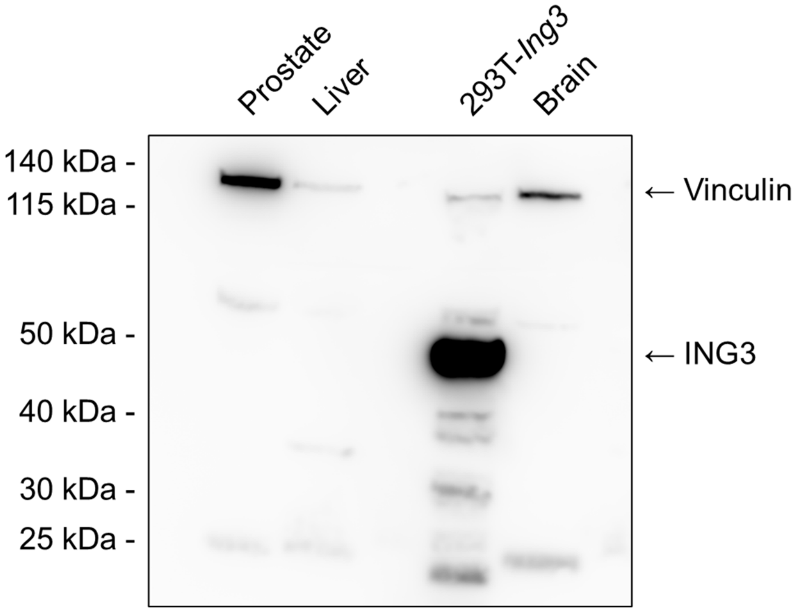

Figure S2. Western blot analysis of ING3 in wildtype mouse tissues. Lysate from HEK 293T cells overexpressing ING3 was used as positive control. Vinculin was used as loading control.

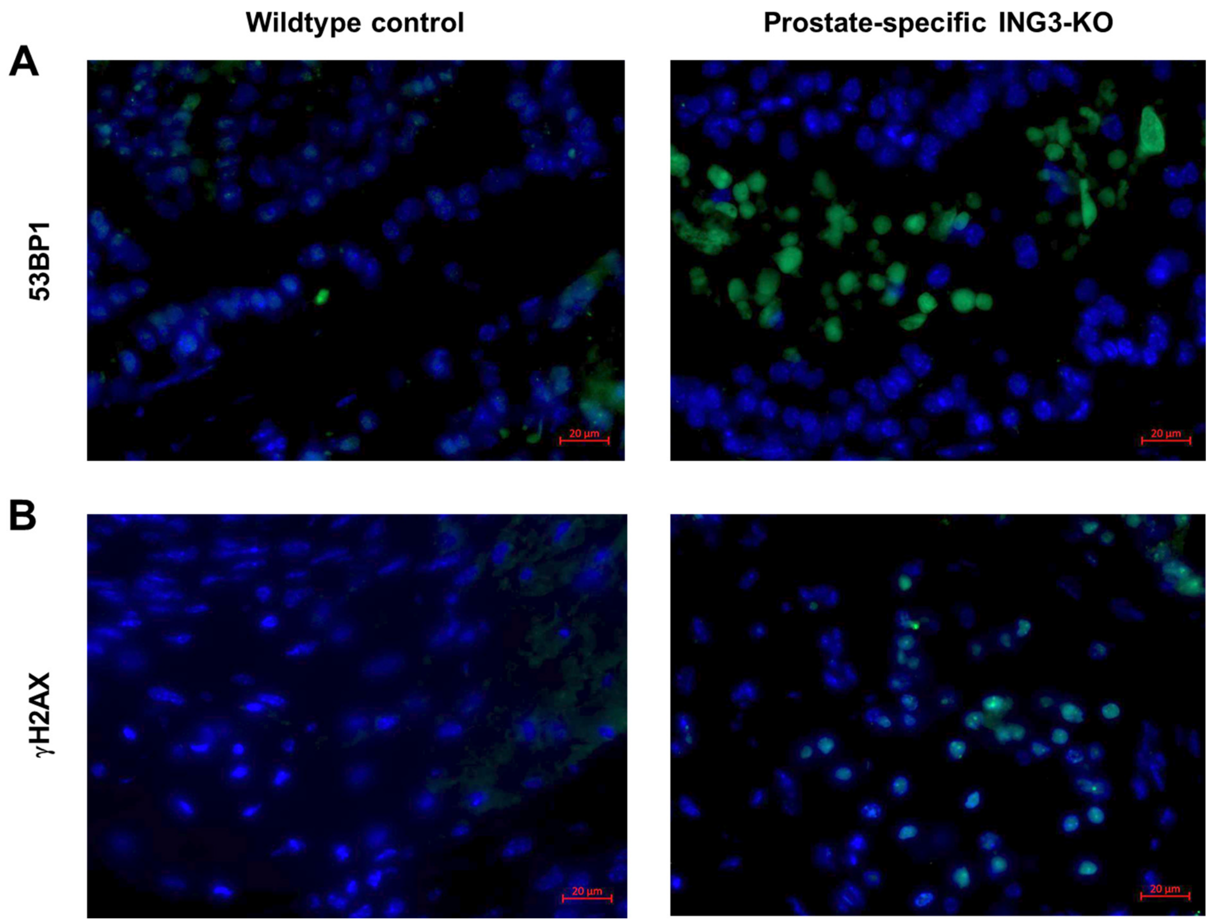

Figure S3. Representative immunofluorescence images of the prostate. (**A**) 53BP1 (green) and (**B**) γH2AX (green) signal intensity is increased upon prostate-specific ING3 ablation. Nuclei were counterstained with DAPI (blue). Left side panel: *Ing3*$^{fl/fl}$; PB-Cre4$^{+/+}$ (*n* = 3 for 53BP1 staining and *n* = 2 for γH2AX staining), right side panel: *Ing3*$^{fl/fl}$; PB-Cre4$^{+/T}$ (*n* = 3). Scale bars are shown at the right bottom of each image.

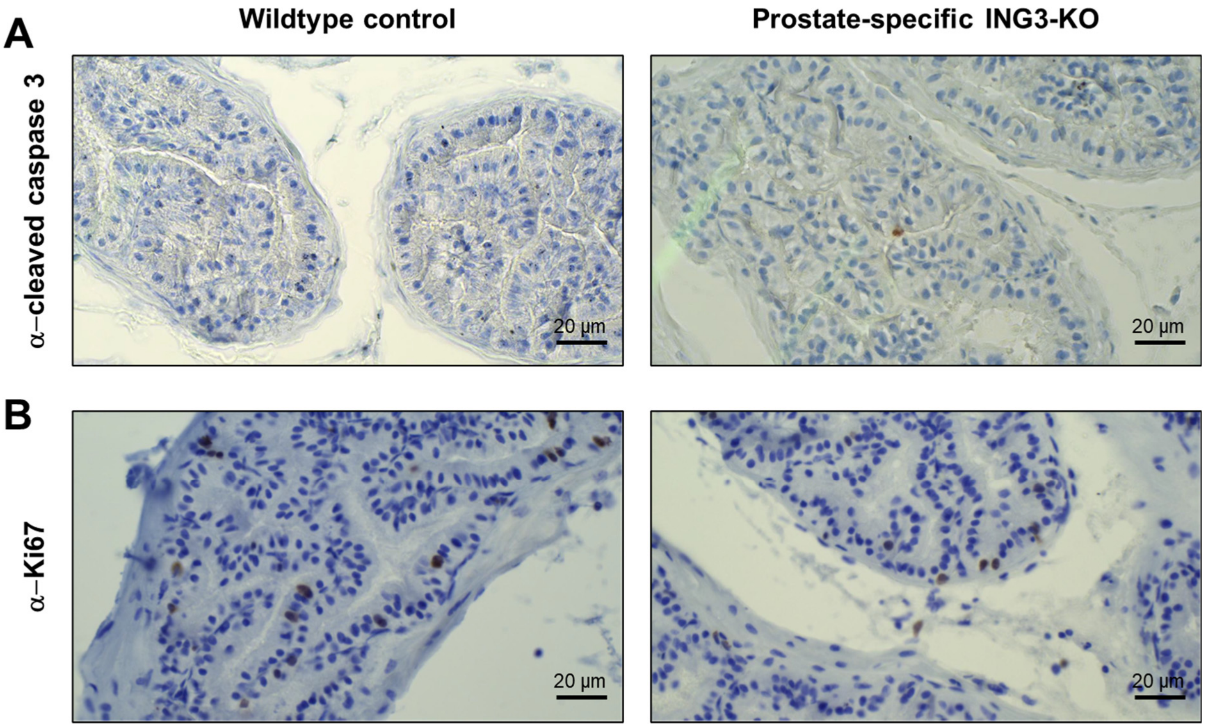

Figure S4. Representative images of cleaved caspase 3 and Ki67 staining of prostatic lobes of age-matched prostate-specific *Ing3* knockouts and wild type controls. (**A**) Cleaved caspase 3 staining. Left side panel: *Ing3*$^{fl/fl}$; PB-Cre4$^{+/+}$ (*n* = 2), right side panel: *Ing3*$^{fl/fl}$; PB-Cre4$^{+/T}$ (*n* = 3). (**B**) Ki67 staining. Left side panel: *Ing3*$^{fl/fl}$; PB-Cre4$^{+/+}$ (*n* = 2), right side panel: *Ing3*$^{fl/fl}$; PB-Cre4$^{+/T}$ (*n* = 2). Magnification 400 ×. Scale bars are shown at the right bottom of each image.

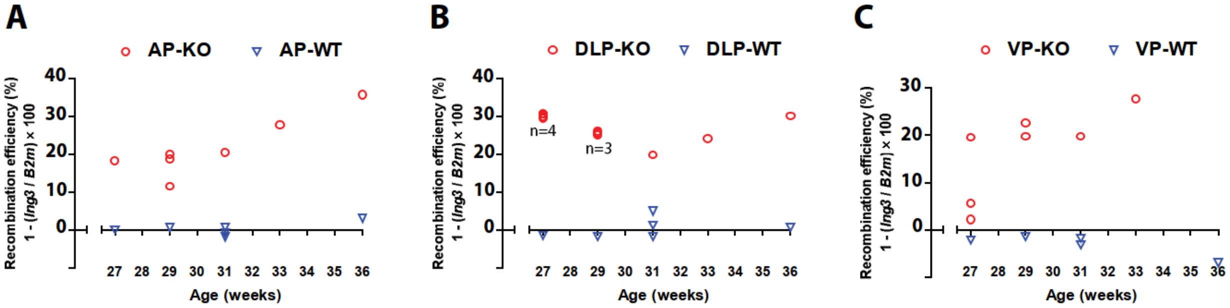

Figure S5. Recombination efficiency in the prostate lobes in prostate-specific knockout and wild type *Ing3* mice as a function of age. (**A**) anterior prostate (AP), (**B**) dorsolateral prostate (DLP), and (**C**) ventral prostate (VP). (KO: knockout (*Ing3*$^{fl/fl}$; PbCre4$^{+/T}$), WT: wild type (*Ing3*$^{fl/fl}$; PbCre4$^{+/+}$), each datapoint represents an individual mouse if not indicated otherwise).

Table S1: Animals used for dPCR calibration.

Animal ID	Sex	*Ing3*
0117-0394	male	Δ/+
0120-0172	female	fl/fl
0120-0173	female	fl/fl
0120-0174	female	fl/fl
0120-0176	female	fl/fl
0149-0460	female	Δ/+
0149-0461	female	Δ/Δ
0149-0462	female	Δ/+
0149-0463	female	Δ/+
0149-0464	female	Δ/+
0149-0465	female	Δ/Δ
0149-0466	male	Δ/Δ
0149-0467	male	Δ/+
0149-0468	male	Δ/Δ

Table S2: Animals used for gross anatomic inspection and prostate-specific recombination efficiency assessment by dPCR.

Animal ID	Sex	*Ing3*	PB-Cre4	Age (weeks)
0149-0417	male	fl/fl	+/+	36
0149-0418	male	fl/fl	+/T	36
0149-0426	male	fl/fl	+/T	33
0149-0451	male	fl/fl	+/+	31
0149-0452	male	fl/fl	+/+	31
0149-0453	male	fl/fl	+/T	31
0149-0454	male	fl/fl	+/+	31
0149-0486	male	fl/fl	+/T	29
0149-0487	male	fl/fl	+/T	29
0149-0488	male	fl/fl	+/+	29
0149-0501	male	fl/m	+/T	48
0149-0503	male	fl/m	+/+	48
0149-0505	male	fl/m	+/+	48
0149-0529	male	fl/m	+/+	40
0149-0530	male	fl/m	+/+	40
0149-0537	male	fl/m	+/T	41
0149-0545	male	fl/m	+/T	37
0149-0546	male	fl/m	+/T	37
0149-0547	male	fl/m	+/+	37
0149-0588	male	fl/fl	+/T	29
0149-0619	male	fl/fl	+/T	27
0149-0620	male	fl/fl	+/T	27
0149-0625	male	fl/fl	+/T	27
0149-0626	male	fl/fl	+/+	27
0149-0627	male	fl/fl	+/T	27

Table S3: Animals used for gross anatomic inspection, immunohistochemistry and immunofluorescence.

Animal ID	Sex	*Ing3*	PB-Cre4	Age (weeks)
0149-0228	male	fl/fl	+/T	87
0149-0277	male	fl/fl	+/T	74
0149-0278	male	fl/fl	+/T	74
0149-0279	male	fl/fl	+/+	74
0149-0304	male	fl/fl	+/+	73
0149-0381	male	fl/fl	+/T	49
0149-0410	male	fl/fl	+/+	35
0149-0413	male	fl/fl	+/+	35
0149-0419	male	fl/fl	+/T	35
0149-0424	male	fl/fl	+/T	32
0149-0425	male	fl/fl	+/+	32
0149-0557	male	fl/fl	+/+	13
0149-0558	male	fl/fl	+/T	13
0149-0574	male	fl/fl	+/+	12
0482-0142	male	+/+	+/+	27

Table S4: Animals used for gross anatomic inspection only.

Animal ID	Sex	*Ing3*	PB-Cre4	Age (weeks)
0149-0305	male	fl/fl	+/+	73
0149-0409	male	fl/fl	+/+	35
0149-0411	male	fl/fl	+/T	35
0149-0412	male	fl/fl	+/+	35
0149-0423	male	fl/fl	+/T	32
0149-0559	male	fl/fl	+/T	13
0149-0565	male	fl/fl	+/T	12
0149-0572	male	fl/fl	+/T	12
0149-0573	male	fl/fl	+/+	12

Table S5: Animals used for X-gal staining of cryosections.

Animal ID	Sex	*Ing3*-LacZ	Age (weeks)
1020-0175	female	+/+	23
1032-1573	male	+/+	38
1055-0657	female	+/LacZ	60
1055-0659	male	+/LacZ	59
1055-0666	male	+/LacZ	46
1055-0672	female	+/LacZ	40

Table S6: Animals used for X-gal staining of whole mount tissues.

Animal ID	Sex	*Ing3*-LacZ	Age (weeks)
0482-0160	male	+/+	59
0482-0161	male	+/+	60
0482-0162	male	+/+	59
1055-0665	male	+/LacZ	46
1055-0667	male	+/LacZ	46
1055-0675	male	+/LacZ	39

Table S7: Instrument readout for dPCR calibration.

Animal ID	1 - (*Ing3*/*B2m*)	Copies/rxn (*Ing3*)	CI Copies/rxn (*Ing3*)	Copies/µl (*Ing3*)	Copies/rxn (*B2m*)	CI Copies/rxn (*B2m*)	Copies/µl (*B2m*)	Qualified partitions	Filled partitions
0117-0394	49.72%	0.356	0.346–0.367	471.79	0.708	0.693–0.725	938.3	15943	18773
0120-0172	-1.16%	0.736	0.719–0.752	974.48	0.727	0.711–0.744	963.31	15689	18180
NTC	93.96%	1.18E-04	2.95E-5–4.72E-4	0.156	1.95E-03	1.39E-3–2.74E-3	2.584	16931	18731
0120-0173	-0.88%	0.472	0.46–0.484	624.73	0.468	0.456–0.479	619.3	16924	18357
0120-0174	-0.71%	0.785	0.768–0.802	1039.8	0.78	0.763–0.797	1032.5	15859	17647
0120-0176	-3.87%	0.724	0.708–0.74	959.01	0.697	0.682–0.713	923.28	16742	17649
0149-0460	49.52%	0.247	0.238–0.256	327.16	0.489	0.476–0.503	648.1	14035	16142
0149-0461	99.91%	4.55E-04	2.17E-4–9.55E-4	0.603	0.526	0.513–0.54	697.15	15380	16556
0149-0462	50.89%	0.3	0.29–0.311	397.67	0.611	0.596–0.628	809.76	13042	15209
0149-0463	48.36%	0.154	0.148–0.16	204.12	0.298	0.29–0.307	395.29	16922	18340
0149-0464	49.36%	0.174	0.168–0.181	230.97	0.344	0.335–0.354	456.08	16563	18171
0149-0465	99.98%	1.19E-04	2.96E-5–4.74E-4	0.157	0.518	0.505–0.53	685.77	16876	17759
0149-0466	99.71%	1.61E-03	1.10E-3–2.37E-3	2.139	0.56	0.547–0.574	741.58	16113	18442
0149-0467	46.57%	0.165	0.157–0.173	218.12	0.308	0.297–0.32	408.23	9954	13911
0149-0468	99.99%	6.13E-05	8.63E-6–4.35E-4	8.11E-02	0.64	0.626–0.655	848.29	16327	17329
NTC	0.00%	5.85E-05	8.23E-6–4.15E-4	7.74E-02	5.85E-05	8.23E-6–4.15E-4	7.74E-02	17108	18122

CI: 95% confidence interval; NTC: no template control; Rxn: reaction

Table S8: Instrument readout for dPCR recombination experiment.

Animal ID/ prostate lobe	1 - (*Ing3*/*B2m*)	Copies/rxn (*Ing3*)	CI Copies/rxn (*Ing3*)	Copies/µl (*Ing3*)	Copies/rxn (*B2m*)	CI Copies/rxn (*B2m*)	Copies/µl (*B2m*)	Qualified partitions	Filled partitions
0149-0417/AP	3.05%	0.51	0.501–0.526	679.97	0.53	0.517–0.542	701.37	17515	18612
0149-0417/DLP	0.64%	0.68	0.668–0.698	904.48	0.69	0.673–0.702	910.28	17346	18241
0149-0417/VP	-7.00%	0.38	0.373–0.394	507.98	0.36	0.349–0.368	474.75	17094	18345
0149-0418/AP	35.68%	0.51	0.501–0.525	679.45	0.80	0.781–0.814	1056.40	17616	18394
0149-0418/DLP	30.20%	0.38	0.365–0.387	497.92	0.54	0.525–0.552	713.40	15571	17440
0149-0418/VP	8.22%	0.05	4.92E-2–5.61E-2	69.54	0.06	5.37E-2–6.09E-2	75.77	17311	18426
0149-0426/AP	27.72%	0.37	0.359–0.38	488.79	0.51	0.498–0.524	676.24	15428	18410
0149-0426/DLP	24.14%	0.43	0.414–0.436	562.69	0.56	0.547–0.574	741.75	16351	18006
0149-0426/VP	27.65%	0.19	0.18–0.193	246.82	0.26	0.25–0.266	341.13	17815	18447
0149-0451/AP	-0.75%	0.61	0.599–0.626	811.47	0.61	0.595–0.622	805.44	17999	18863
0149-0451/DLP	-1.79%	0.53	0.518–0.543	702.22	0.52	0.509–0.533	689.90	17371	18898
0149-0451/VP	-3.20%	0.40	0.391–0.412	531.40	0.39	0.379–0.399	514.90	17718	18501
0149-0452/AP	0.74%	1.15	1.125–1.169	1518.60	1.16	1.133–1.178	1529.90	17670	18386
0149-0452/DLP	4.86%	0.62	0.607–0.635	822.30	0.65	0.638–0.667	864.28	17973	18609
0149-0452/VP	-1.84%	0.60	0.582–0.609	788.19	0.58	0.571–0.598	773.92	17201	18359
0149-0453/AP	20.42%	0.54	0.528–0.554	716.78	0.68	0.665–0.695	900.76	16960	18230
0149-0453/DLP	19.75%	0.35	0.342–0.361	465.57	0.44	0.427–0.449	580.16	17289	18653
0149-0453/VP	19.78%	0.46	0.448–0.472	609.05	0.57	0.56–0.587	759.22	16845	17899
0149-0454/AP	-1.87%	0.85	0.835–0.871	1129.50	0.84	0.819–0.855	1108.80	15832	18029
0149-0454/DLP	1.19%	0.47	0.459–0.483	623.33	0.48	0.464–0.489	630.82	16296	18156
0149-0454/VP	-9.26%	0.13	0.121–0.132	167.74	0.12	0.111–0.121	153.52	16314	17901
0149-0486/AP	19.92%	0.45	0.436–0.459	593.06	0.56	0.546–0.573	740.61	16386	18230
0149-0486/DLP	25.04%	1.09	1.073–1.116	1449.20	1.46	1.432–1.488	1933.40	17166	18606
0149-0486/VP	22.54%	0.30	0.29–0.308	395.31	0.39	0.375–0.396	510.33	16718	18270
0149-0487/AP	11.50%	0.41	0.394–0.416	536.16	0.46	0.446–0.469	605.84	15951	17965
0149-0487/DLP	26.15%	0.32	0.313–0.332	426.79	0.44	0.425–0.448	577.89	16991	18389

Animal ID/ prostate lobe	1 - (*Ing3*/*B2m*)	Copies/rxn (*Ing3*)	CI Copies/rxn (*Ing3*)	Copies/µl (*Ing3*)	Copies/rxn (*B2m*)	CI Copies/rxn (*B2m*)	Copies/µl (*B2m*)	Qualified partitions	Filled partitions
0149-0487/VP	27.51%	1.77	1.734–1.807	2343.90	2.44	2.387–2.496	3233.30	16115	17670
0149-0488/AP	0.55%	0.83	0.81–0.845	1095.70	0.83	0.814–0.85	1101.80	16275	17838
0149-0488/DLP	-1.78%	0.64	0.622–0.651	842.55	0.63	0.611–0.639	827.82	17038	18558
0149-0488/VP	-1.43%	0.25	0.238–0.254	325.26	0.24	0.234–0.25	320.66	16602	18340
0149-0501/AP	6.01%	0.97	0.945–0.985	1278.50	1.03	1.006–1.048	1360.20	15950	18169
0149-0501/DLP	12.69%	0.22	0.211–0.227	290.42	0.25	0.243–0.26	332.64	14617	15531
0149-0501/VP	4.42%	0.56	0.545–0.574	740.58	0.59	0.57–0.6	774.82	14280	15685
0149-0503/AP	-1.07%	0.52	0.504–0.53	684.78	0.51	0.499–0.524	677.53	16038	18010
0149-0503/DLP	-0.56%	2.05	2.006–2.087	2710.00	2.04	1.994–2.076	2694.80	16095	17847
0149-0503/VP	-1.97%	0.27	0.264–0.281	360.77	0.27	0.259–0.276	353.79	17192	18278
0149-0505/AP	-0.89%	0.58	0.567–0.594	768.94	0.58	0.562–0.589	762.16	16088	18263
0149-0505/DLP	-1.60%	0.57	0.556–0.583	753.90	0.56	0.547–0.574	742.06	16421	18086
0149-0505/VP	-1.10%	0.81	0.79–0.824	1068.20	0.80	0.781–0.815	1056.60	16948	18370
0149-0529/AP	-0.78%	0.66	0.641–0.672	869.15	0.65	0.636–0.666	862.44	15683	18266
0149-0529/DLP	-1.00%	0.59	0.572–0.601	776.68	0.58	0.567–0.595	769.01	15588	17427
0149-0529/VP	-8.11%	0.27	0.257–0.274	351.37	0.25	0.237–0.254	325.00	16249	18330
0149-0530/AP	-0.86%	0.63	0.614–0.649	835.61	0.63	0.608–0.643	828.48	11231	13412
0149-0530/DLP	1.30%	0.71	0.696–0.727	941.75	0.72	0.705–0.736	954.16	17221	18423
0149-0530/VP	-6.87%	0.42	0.405–0.428	551.40	0.39	0.379–0.4	515.96	16177	18168
0149-0537/AP	15.19%	2.22	2.178–2.272	2945.90	2.62	2.565–2.682	3473.70	16360	17573
0149-0537/DLP	28.93%	0.27	0.262–0.281	359.40	0.38	0.37–0.394	505.72	12835	17670
0149-0537/VP	-1.21%	0.20	0.196–0.212	269.56	0.20	0.193–0.209	266.34	14108	15929
0149-0545/AP	9.80%	1.10	1.078–1.122	1456.80	1.22	1.196–1.243	1615.00	16986	18415
0149-0545/DLP	12.71%	0.55	0.534–0.561	724.66	0.63	0.612–0.642	830.21	15036	16335
0149-0545/VP	-2.05%	0.38	0.373–0.393	506.69	0.38	0.365–0.385	496.52	17559	18238
0149-0546/AP	18.50%	0.73	0.709–0.741	960.47	0.89	0.871–0.909	1178.50	16218	17173
0149-0546/DLP	19.91%	0.54	0.527–0.555	716.91	0.68	0.66–0.692	895.18	14228	15905

Animal ID/ prostate lobe	1 - (*Ing3*/*B2m*)	Copies/rxn (*Ing3*)	CI Copies/rxn (*Ing3*)	Copies/µl (*Ing3*)	Copies/rxn (*B2m*)	CI Copies/rxn (*B2m*)	Copies/µl (*B2m*)	Qualified partitions	Filled partitions
0149-0546/VP	13.34%	0.33	0.321–0.34	438.14	0.38	0.372–0.392	505.57	17066	18317
0149-0547/AP	0.65%	0.76	0.746–0.78	1009.90	0.77	0.75–0.785	1016.50	15251	16808
0149-0547/DLP	-2.54%	0.42	0.412–0.434	560.26	0.41	0.402–0.424	546.36	16298	18018
0149-0547/VP	-0.22%	0.28	0.275–0.293	375.87	0.28	0.275–0.292	375.03	16893	17993
0149-0588/AP	18.75%	0.65	0.632–0.661	856.07	0.80	0.779–0.812	1053.60	16773	18233
0149-0588/DLP	25.57%	0.44	0.425–0.448	578.00	0.59	0.572–0.601	776.57	15602	17225
0149-0588/VP	19.78%	0.19	0.187–0.201	256.82	0.24	0.234–0.25	320.16	15244	18216
0149-0619/AP	18.24%	0.91	0.892–0.93	1206.20	1.11	1.092–1.136	1475.30	16881	17710
0149-0619/DLP	29.57%	0.33	0.316–0.337	432.29	0.46	0.451–0.477	613.78	13710	15661
0149-0619/VP	2.14%	0.28	0.272–0.289	371.51	0.29	0.278–0.296	379.65	16282	17850
0149-0620/AP	8.55%	5.84	5.572–6.124	7736.70	6.39	6.039–6.755	8459.80	16707	18387
0149-0620/DLP	30.62%	0.60	0.585–0.613	793.34	0.86	0.846–0.881	1143.40	17100	18472
0149-0620/VP	19.55%	0.55	0.536–0.562	726.77	0.68	0.667–0.697	903.35	16472	18293
0149-0625/AP	35.30%	1.93	1.89–1.963	2551.30	2.98	2.913–3.043	3943.30	17296	18379
0149-0625/DLP	30.71%	0.54	0.527–0.553	714.54	0.78	0.762–0.796	1031.20	16357	18257
0149-0625/VP	17.70%	0.13	0.124–0.137	173.07	0.16	0.152–0.166	210.29	12646	15198
0149-0626/AP	0.05%	1.28	1.252–1.302	1691.30	1.28	1.253–1.303	1692.20	16545	18138
0149-0626/DLP	-1.53%	1.07	1.045–1.088	1412.60	1.05	1.03–1.072	1391.30	16548	18215
0149-0626/VP	-2.17%	0.77	0.749–0.781	1013.00	0.75	0.733–0.765	991.45	17101	18340
0149-0627/AP	23.16%	1.36	1.333–1.386	1800.40	1.77	1.734–1.804	2343.00	16170	18033
0149-0627/DLP	29.99%	0.60	0.587–0.615	795.99	0.86	0.841–0.876	1136.90	16724	17915
0149-0627/VP	5.60%	0.44	0.429–0.452	582.84	0.47	0.454–0.478	617.41	15700	17699
NTC	-600.48%	0.00	2.09E-4–9.18E-4	0.58	0.00	8.81E-6–4.44E-4	0.08	15995	17953
NTC	0.00%	0.00	1.22E-4–7.04E-4	0.39	0.00	1.22E-4–7.04E-4	0.39	17074	17703

CI: 95% confidence interval; NTC: no template control; Rxn: reaction; AP: anterior prostate; DLP: dorsolateral prostate; VP: ventral prostate; Grey highlight: chip excluded from statistical analysis (copy count out of dynamic range)
